# Patient self-reported concerns in inflammatory bowel diseases: A gender-specific subjective quality-of-life indicator

**DOI:** 10.1371/journal.pone.0171864

**Published:** 2017-02-10

**Authors:** Valérie Pittet, Carla Vaucher, Florian Froehlich, Bernard Burnand, Pierre Michetti, Michel H. Maillard, On behalf of the Swiss IBD Cohort Study Group

**Affiliations:** 1 Institute of Social & Preventive Medicine (IUMSP), Lausanne University Hospital, Lausanne, Switzerland; 2 Division of Gastroenterology & Hepatology, Lausanne University Hospital, Lausanne, Switzerland; 3 Division of Gastroenterology & Hepatology, Basel University Hospital, Basel, Switzerland; 4 Crohn and Colitis Center, Clinique La Source-Beaulieu, Lausanne, Switzerland; University Hospital Llandough, UNITED KINGDOM

## Abstract

**Background:**

Patient-reported disease perceptions are important components to be considered within a holistic model of quality of care. Gender may have an influence on these perceptions. We aimed to explore gender-specific concerns of patients included in a national bilingual inflammatory bowel disease cohort.

**Methods:**

Following a qualitative study, we built a questionnaire comprising 37 items of concern. Answers were collected on a visual analog scale ranging from 0 to 100. Principal axis factor analysis was used to explore concern domains. Linear multiple regressions were conducted to assess associations with patient characteristics.

**Results:**

Of 1102 patients who replied to the survey, 54% were female and 54% had Crohn’s disease. We identified six domains of concern: socialization and stigmatization, disease-related constraints and uncertainty, symptoms and their impact on body and mind, loss of body control (including sexuality), disease transmission, and long-term impact of the disease. Cancer concerns were among the highest scored by all patients (median 61.8). Severity of symptoms was the only factor associated with concerns, unrelated to dimension and gender (p<0.015). In women, being >40 years decreased disease-related constraints and uncertainty concerns, and being at home or unemployed increased them. Treatments were associated with increased socialization and stigmatization and with increased disease-related constraints and uncertainty concerns in men. Overall, psychosomatic characteristics were highly associated with concerns for both men and women. Depending on the concern dimensions, increased levels of concern were associated with the highest signs of anxiety in women or depression in men, as well as lower health-related quality of life in men.

**Conclusions:**

Patients have numerous concerns related to their illness that need to be reassessed regularly. Concerns differ between men and women, suggesting that information and communication about the disease should take gender differences and subjective perceptions of quality of life into consideration.

## Introduction

Patient-reported perceptions of quality of life (QoL) are important components to consider because of their potential influence on disease self-management and thus on health outcomes [1,2]. Compared with objective clinical assessments, subjective considerations of the impact of the disease and its treatments are viewed with some skepticism [3] by clinicians, although individual well-being might be influenced by, e.g., both symptoms and concerns about potential symptoms. In addition, positive dimensions of QoL are more easily taken into consideration than negative dimensions such as worries and concerns regarding the impact of the disease, although these are of equal importance for assessing a patient’s overall well-being [4]. Indeed, concerns might reflect how conscious the patient is of the disease, thus indicating areas for knowledge exchange and communication improvements with clinicians.

Studies on concerns of people with inflammatory bowel disease (IBD) have been conducted since the early 1990s when the Rating Form of IBD Patient Concerns (RFIPC) questionnaire [5] was developed and validated among a patient association population [6], the largest survey conducted until now. The RFIPC was used from 1995 to 2012 to assess concerns of smaller samples of patients from diverse countries that were generally formed by consecutive enrollment of consenting patients in clinical centers [1,7–16]. However, concerns can evolve over time. Putative reasons for dynamic changes in concerns include improvements in treatment strategies and in disease- or treatment-related information given to patients over time. One study investigated changes in concerns over time [14], but the period between assessments was too short to measure clear differences. In addition, concerns can differ by gender, as women may perceive symptoms differently than men, due to, e.g., menstrual cycles[17,18], and express specific coping strategies and perspectives toward living with IBD[19] that may have an impact on diet[20,21], communication with physicians[22], socialization, or sexual health[23,24].

The aims of the present study were to explore patients’ current concerns, to survey concerns of a large number of patients included in a national IBD cohort, and to assess gender-specific concerns and their associated factors.

## Methods

### Study design

We undertook a mixed-methods study using an exploratory sequential design. We first conducted two focus group discussions with 14 IBD patients to explore treatments, disease-related concerns, and expectations. We followed recommendations[25] stating that these discussions should involve 6 to 12 participants. Detailed patient selection criteria were previously described[26]. Focus group discussions were audio-recorded and transcribed for research purposes with the participants’ written consent. Content analyses were performed to explore main categories of concerns being addressed. This allowed us to list additional concerns beyond those already used in the RFIPC developed in 1991[5]. Moreover, we grouped four items used in the RFIPC by combining pairs of items, in keeping with the way they were expressed by patients: i.e., “feeling dirty or smelly” and “producing unpleasant odors” was labeled “feeling dirty or smelly or producing unpleasant odors,” and “attractiveness” and “feelings about my body” was labeled “attractiveness.” We thus built a 37-item questionnaire to conduct the survey. Answers to the question, “Because of your condition, how concerned are you with…?” were collected on a visual analog scale ranging from 0 to 100 (0 = not at all, 100 = a great deal).

### Cohort data

The questionnaire was sent to all adult patients with active follow-up enrolled in the Swiss IBD Cohort (SIBDC) by January 2015. To characterize patients, we extracted data from SIBDC databases. Clinical characteristics were collected by gastroenterologists or trained study nurses during medical visits.

### Statistical analysis

Descriptive analyses that included numbers and percentages were performed to characterize the study population. The mean and median (interquartile range) were calculated for each concern, as well as the mean (SD) for the sum score. Missing values were replaced by a score of 50, in accordance with the instructions given to patients: “If you have no opinion or are undecided, please put a cross in the middle of the scale.” Means were added to allow international comparisons, although all measures were non-normally distributed. We used the Wilcoxon rank-sum test to test distribution differences of the concerns (statistical significance: p-value <0.0014, with Bonferroni correction for multiple testing); t-tests were performed to test for mean differences of the sum scores (statistical significance: p-value <0.05). A factor analysis using the principal axis factor method was conducted to explore the dimensions of the main concerns[27] because individual concern measures were non-normally distributed. We retained factors with correlation matrix eigenvalues ≥1. The principal axis factor was first conducted with all of the individual concern items, and then the model was respecified by deleting one item (“ability to have children”) because of its low communality. Varimax rotation with the Kaiser normalization method was performed, and we retained factors with loadings ≥0.35[28]. Cronbach’s alphas were calculated to assess the internal consistency of items in each dimension. For the first four dimensions (i.e., comprising more than two items), we calculated a non-weighted sum score of each item. We conducted multiple linear regressions, stratified by gender, to assess associations between concern dimensions and patient characteristics.

We checked associations between concerns and the following variables: type of diagnosis (Crohn’s disease, ulcerative colitis, IBD—undetermined), Crohn’s disease location (ileal, ileocolic, colonic only, upper gastrointestinal) and phenotype (inflammatory, stricturing, penetrating, perianal involvement), ulcerative colitis extension (proctitis, left-sided, pancolitis), history of extraintestinal manifestations and resection surgery (yes/no), highest treatment line achieved (no treatment, 5-aminosalicylic acid compounds or steroids, immunomodulators, biologicals), and disease duration. Patient self-reported characteristics, collected through paper questionnaires, included the following: gender, age, language of questionnaire completion (French/German), education level (none or compulsory/secondary education [professional/general], upper secondary education/tertiary education), working status (employed/in training/at home or unemployed/retired or annuitant). Finally, we assessed the association between concerns and other health-related QoL measures. We used the SF-36 questionnaire, divided into two subscores: the Physical Component Summary and the Mental Component Summary. The Inflammatory Bowel Disease Questionnaire (IBDQ) comprises 32 items and was assessed with four subscores (bowel symptoms, systemic symptoms, emotional function, and social function). Mood was assessed through the Hospital Anxiety and Depression Scale, which was divided into two subscales, one assessing depression and the other anxiety; coping was assessed with the Coping Inventory for Stressful Situations [29].

Concern dimensions were normalized by using power transformations. This implied that crude multiple linear regression coefficients might be difficult to interpret or compare. For this reason, results were reported with signs and significance of associations only. To properly separate the effect of anxiety and depression from the effect of QoL measures, we first conducted a linear regression with QoL measures as dependent variables and anxiety and depression scores as explanatory variables. The residuals of these regressions, i.e., QoL measures from which the anxiety and depression shared components were removed, were used in the multiple linear regressions as explanatory variables. To construct multiple linear regression models, we performed the following steps: (1) We assessed all covariates in univariate linear regression models; (2) all covariates with a p-value <0.20 were entered into a multiple linear regression model; (3) nonsignificant covariates were excluded one by one until all remaining covariates were statistically significant (p-value <0.05); (4) nonsignificant covariates were added one by one and kept in the model if they became significant until a final model could be determined, while we checked for model consistency.

Factor analyses were conducted with SPSS Statistics 23 (IBM Corp., New York, NY, USA). Descriptive and regression analyses were conducted by using STATA statistical software v.14.1 (STATA Corp., College Station, TX, USA).

### Ethics approval

Ethics approval was obtained from the regional Swiss Ethics Committees in which cohort participants were enrolled (Commission d’éthique du Canton de Vaud/Protocol no. 33/06). Ethics approval was obtained to conduct focus groups (Commission d’éthique du Canton de Vaud /Protocol no. 185/13).

## Results

### Newly expressed concerns

Analysis of focus group discussion content yielded 14 additional concerns expressed by patients. These concerns were related to support given by relatives, having to talk about the disease with relatives, professional future, diet, having to take life-long treatments, the chronicity of the disease, being addicted to medications, the origins of the disease, fatigue, the influence of the disease on physical and sports performance, the link between stress and disease, the difficulty in predicting relapses, the ability of children to develop the same disease, and medical consultations and exams.

### Characteristics of the sample population

Among the 2094 patients who actively participated in follow-up and agreed to regularly receive self-reported questionnaires, 1123 (54%) replied and 1102 could be used in the study. Women responded more frequently than did men (54% vs. 46%). Socio-demographic variables were significantly different between men and women (Table 1). Men were older than women (48.7 vs. 46.5 years, respectively; p = 0.017) and had a higher educational level. Indeed, 44.6% of men had at least an upper secondary education, compared with 27.3% of women (p<0.001), and a higher proportion of women than men were at home or unemployed (3.8% vs. 14.8%, respectively; p<0.001). Overall, clinical characteristics and treatments were similar for men and women, except for extraintestinal manifestations, a history of which was more frequently found in women than in men (58.0% vs. 47.2%, respectively; p<0.001). About one third of women presented with signs of anxiety, compared with one fifth of men (p<0.001). Signs of depression were noticed in one seventh of all responders, with no difference between genders. General and IBD-specific health-related QoL was lower among women than among men.

**Table 1:** Baseline patient characteristics. Values are number and percentages unless otherwise specified.

Variables	All (N)	Men	Women	p-value
**All**	1102	504 (45.7)	598 (54.3)	
**German speakers**	761	365 (72.4)	396 (66.2)	0.027
Age*	47.5 (15.2)	48.7 (15.7)	46.5 (14.7)	0.017
**Education level**				<0.001
*None or compulsory*	100	37 (7.9)	63 (11.0)	
*Secondary education (professional)*	411	165 (35.0)	246 (43.0)	
*Secondary education (general)*	166	59 (12.5)	107 (18.7)	
*Upper secondary education*	214	123 (26.1)	91 (15.9)	
*Tertiary education*	152	87 (18.5)	65 (11.4)	
**Working status**				<0.001
*Employed*	743	357 (74.8)	386 (68.7)	
*In training*	30	13 (2.7)	17 (3.0)	
*At home/unemployed*	101	18 (3.8)	83 (14.8)	
*Retired/annuitant*	165	89 (18.7)	76 (13.5)	
Age at diagnosis*	31.8 (13.8)	33.2 (14.4)	30.6 (13.2)	0.003
Disease duration*	15.7 (10.2)	15.6 (10.3)	15.9 (10.1)	0.518
**Diagnosis**				0.027
*CD*	596	256 (50.8)	340 (56.9)	
*UC*	475	228 (45.2)	247 (41.3)	
*IBDU*	31	20(4.0)	11 (1.8)	
**Disease location (CD)**				0.320
*Ileal*	186	86 (33.6)	100 (29.4)	
*Colonic*	209	93 (36.3)	116 (34.1)	
*Ileocolonic*	168	65 (25.4)	103 (30.3)	
*Upper GI only*	14	7 (2.7)	7 (2.1)	
**Disease behavior (CD)**				0.325
*Inflammatory*	294	118 (46.1)	176 (51.8)	
*Stricturing*	198	88 (34.4)	110 (32.3)	
*Penetrating*	104	50 (19.5)	54 (15.9)	
**Disease extension (UC)**				0.115
*Proctitis*	117	48 (19.3)	69 (26.7)	
*Left-sided colitis*	207	113 (45.6)	94 (36.4)	
*Pancolitis*	172	83 (33.5)	89 (34.5)	
**Perianal disease**	235	106 (21.0)	129 (21.6)	0.827
**Highest treatment line**				0.891
*No treatment*	14	5 (1.0)	9 (1.5)	
*5-ASA or steroids*	232	105 (20.8)	127 (21.2)	
*Immunomodulators*	340	157 (31.1)	183 (30.6)	
*Biologicals*	516	237 (47.0)	279 (46.7)	
**History of resection surgery**	287	135 (26.8)	152 (25.4)	0.606
**History of extraintestinal manifestations**	585	238 (47.2)	347 (58.0)	<0.001
**Signs of anxiety**				<0.001
*None*	761	376 (78.2)	385 (65.8)	
*Mild to severe*	305	105 (21.8)	200 (34.2)	
**Signs of depression**				0.663
*None*	903	410 (85.2)	493 (84.3)	
*Mild to severe*	163	71 (14.8)	92 (15.7)	
SF36 physical component*	49.4 (9.2)	51.0 (8.1)	48.2 (9.9)	<0.001
SF36 mental component*	46.6 (10.6)	48.3 (9.7)	45.1 (11.1)	<0.001
IBDQ sum score*	57.7 (9.8)	59.7 (8.8)	56.1 (10.2)	<0.001

* Mean (SD).

CD, Crohn’s disease; UC, ulcerative colitis; IBDU, inflammatory bowel disease—undetermined; GI, gastrointestinal; 5-ASA, 5-aminosalicylic acid; IBDQ, Inflammatory Bowel Disease Questionnaire.

### Levels of individual concerns

Overall, the five most important concerns were loss of bowel control (median score: 70, interquartile range: 38–92), the risk of developing cancer (69, 44–88), the link between stress and disease (68, 44–86), the chronicity of the disease (66, 43–85), and fatigue (65, 31–88) (Table 2). The overall level of concern of patients was 45.3.

**Table 2:** Mean, median, and interquartile range (IQR) scores for each concern according to gender, type of diagnosis, language, and age.

Dimension	Mean	Median (IQR)
Loss of bowel control	61.8	70 (38–92)
Developing cancer	61.1	69 (44–88)
Link between stress and disease	60.3	68 (44–86)
Chronicity of the disease	60.1	66 (43–85)
Fatigue	58.5	65 (31–88)
Having to take life-long treatments	55.8	64 (23–87)
Uncertain nature of the disease	57.7	63 (34–85)
Treatment side effects	54.5	63 (20–84)
Difficulty in predicting relapses	54.6	58 (28–82)
Ability of children to develop the same disease	55.7	58 (25–88)
Having surgery	54.2	57 (20–86)
Energy level	53.1	56.5 (21–79)
Having an ostomy bag	51.9	52 (14–87)
Being addicted to medications	49.5	52 (14–81)
Pain	49.3	51 (19–76)
Influence of the disease on physical and sports performance	47.7	51 (15–74)
Origins of the disease	47.4	51 (13–76)
Ability to achieve full potential	45.3	49 (12–71)
Feeling out of control	43.8	47 (8–75)
Impact on sexual life	43.6	47 (10–70)
Loss of sexual drive	40.3	44 (6–67)
Diet	41.5	43.5 (9–68)
Medical consultations and exams	40.7	41 (11–67)
Intimacy	38.7	37.5 (7–62)
Dying early	39.4	35 (8–65)
Support by relatives	36.1	34 (6–54)
Being a burden on others	39.2	32 (6–69)
Professional future	36.9	29 (6–62)
Having access to quality medical care	37.7	28 (7–66)
Attractiveness	35.9	28 (5–62)
Feeling alone	33.0	19.5 (5–54)
Feeling "dirty" or "smelly" or producing unpleasant odors	34.1	19 (4–62)
Financial difficulties	30.4	16 (4–52)
Ability to have children	30.3	15 (3–50)
Being treated as different	27.1	14 (4–49)
Having to talk about disease with relatives	27.3	14 (4–49)
Passing disease on to others	32.9	14 (3–57)
**Sum score**	45.3	

### Main dimensions of concerns

The principal axis factor yielded six main concern dimensions, which were labeled according to the item(s) with the strongest factor loading (Table 3), namely: (1) socialization and stigmatization, (2) constraints and uncertainty, (3) impact of the disease on body and mind (including symptoms), (4) loss of body control (including sexuality), (5) disease transmission, and (6) long-term impact of the disease. Cronbach’s alpha for the items included in each dimension varied between 0.71 and 0.89. All dimensions explained 48.4% of the common variance. Two items (“ability to have children” and “medical consultations and exams”) had factors loadings that were too low and therefore could not be associated with any dimension.

**Table 3:** Results of the rotated factor matrix.

	Dimensions					
Concerns	Socialization and stigmatization	Constraints and uncertainty	Symptoms (impact on body and mind)	Loss of body control (including sexuality)	Disease transmission	Long-term impact
Feeling alone	**0.64**	0.10	0.25	0.18	0.07	0.20
Being treated as different	**0.63**	0.11	0.14	0.23	0.07	0.19
Intimacy	**0.58**	0.14	0.25	**(0.39)**	0.16	0.11
Support by relatives	**0.52**	0.16	0.26	0.15	0.10	0.11
Being a burden on others	**0.51**	0.19	0.33	0.28	0.12	0.01
Professional future	**0.50**	0.23	**(0.37)**	0.12	0.01	0.06
Financial difficulties	**0.50**	0.22	0.27	0.05	0.04	0.02
Having access to quality medical care	**0.49**	0.23	0.12	0.08	0.16	0.18
Having to talk about disease with relatives	**0.49**	0.21	0.03	0.12	-0.05	0.01
Feeling "dirty" or "smelly" or producing unpleasant odors	**0.48**	0.02	0.12	**(0.45)**	0.13	0.14
Attractiveness	**0.42**	0.20	0.31	0.24	0.21	0.04
Diet	**0.35**	0.32	0.26	0.19	0.08	0.00
Having to take life-long treatments	0.18	**0.70**	0.09	0.09	0.09	0.09
Chronicity of the disease	0.16	**0.65**	0.19	0.27	0.03	0.07
Treatment side effects	0.14	**0.57**	0.19	0.02	0.04	0.11
Uncertain nature of the disease	0.17	**0.56**	0.17	0.32	0.13	0.27
Being addicted to medications	0.15	**0.55**	0.13	0.07	0.13	0.19
Origins of the disease	0.19	**0.38**	0.12	0.12	0.19	0.30
Energy level	0.25	0.17	**0.73**	0.25	0.10	0.13
Fatigue	0.25	0.28	**0.63**	0.19	0.14	0.05
Ability to achieve full potential	**(0.36)**	0.21	**0.62**	0.13	0.04	0.23
Influence of the disease on physical and sports performance	0.30	0.22	**0.50**	0.24	0.10	0.10
Pain	0.34	**(0.36)**	**0.37**	0.21	0.14	0.02
Link between stress and disease	0.23	0.33	**0.35**	0.15	0.12	0.17
Loss of bowel control	0.21	0.30	0.24	**0.59**	0.14	0.10
Feeling out of control	**(0.43)**	0.14	0.19	**0.53**	0.13	0.16
Impact on sexual life	**(0.39)**	0.17	0.33	**0.52**	0.11	-0.04
Difficulty in predicting relapses	0.21	**(0.47)**	0.28	**0.47**	0.14	0.11
Loss of sexual drive	**(0.35)**	0.07	0.32	**0.45**	0.15	0.12
Having surgery	0.20	**(0.40)**	0.11	**0.44**	0.12	0.26
Having an ostomy bag	0.16	0.17	0.14	**0.43**	0.18	0.31
Passing disease on to others	0.16	0.10	0.09	0.14	**0.79**	0.18
Ability of children to develop the same disease	0.06	0.22	0.14	0.20	**0.59**	0.12
Developing cancer	0.08	**(0.38)**	0.10	0.24	0.14	**0.58**
Dying early	0.22	0.26	0.14	0.10	0.11	**0.55**
Medical consultations and exams	0.31	0.34	0.11	0.07	0.09	0.08
Ability to have children	-	-	-	-	-	**-**
**% of total variance explained**	**12.9**	**10.6**	**8.8**	**8.2**	**4.0**	**3.9**
**Cronbach’s alpha**	**0.89**	**0.82**	**0.85**	**0.86**	**0.72**	**0.71**

### Specific gender-related concerns

**Fig 1:**
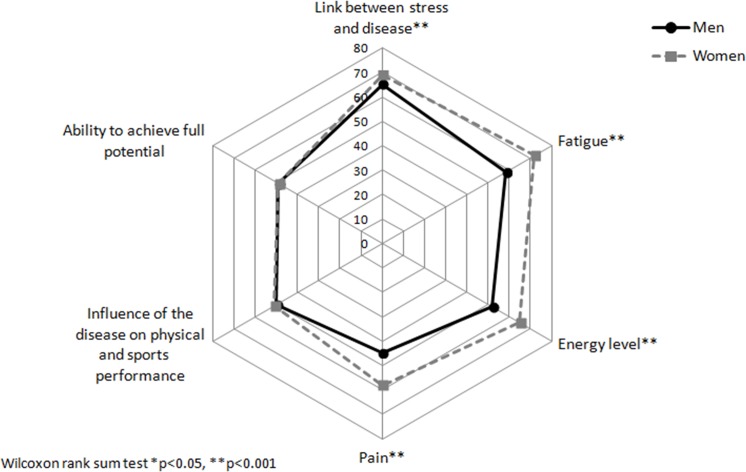
Median for individual concerns related to symptoms according to gender.

**Fig 2:**
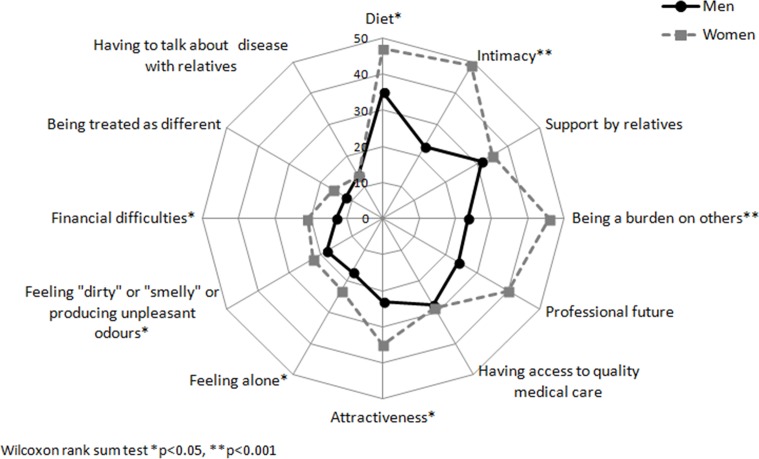
Median for individual concerns related to socialization and stigmatization according to gender.

**Fig 3:**
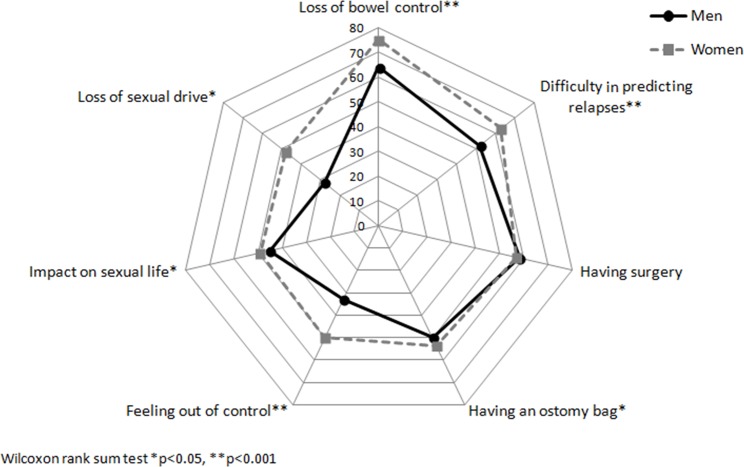
Median for individual concerns related to loss of body control according to gender.

**Fig 4:**
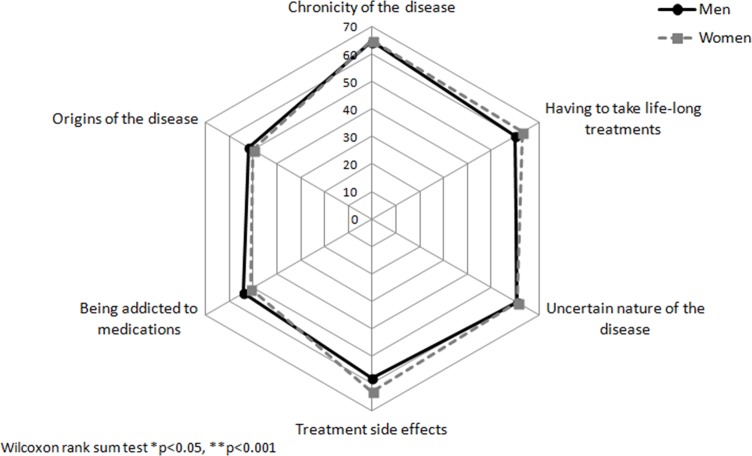
Median for individual concerns related to constraints and uncertainty according to gender.

When we compared the unadjusted median of specific concerns in the four main dimensions to identify where the differences might be, we observed that women felt significantly more concerned by symptoms such as fatigue, pain, and the influence of stress on disease activity and energy level (Fig 1). Significant gender-related differences were observed for personal concerns such as intimacy (Fig 2) and disease-related disabilities, including loss of bowel control, feeling out of control, or being unable to predict relapses (Fig 3). Finally, women seemed more concerned by the burden related to medical consultations and exams, as well as by the social impact of living with the disease. Constraints and uncertainty concerns were not gender specific (Fig 4). Women had significantly higher overall levels of concern than did men (sum score: 47.5 vs. 42.8, respectively, p<0.001)

### Factors associated with concerns

Symptom severity was the only factor associated with increased concerns, regardless of the dimension (Table 4). Education level and disease location or phenotypic characteristics were not associated with any concern dimension. Overall, factors associated with concern dimensions differed according to gender and dimension. Socio-demographic characteristics had an effect on the level of some concerns for women and disease-related characteristics for men. Overall, psychosomatic characteristics were highly associated with concerns of both men and women. Increased levels of concern were associated with the highest signs of anxiety or depression and with lower health-related QoL.

**Table 4:** Multiple linear regressions for each concern dimension stratified by gender. Values indicate the direction of the association and the p-value.

	Socialization and stigmatization		Constraints and uncertainty		Symptoms (impact on body and mind)		Loss of body control (including sexuality)	
	Men	Women	Men	Women	Men	Women	Men	Women
**Socio-demographic characteristics**								
Age >40 years				- / 0.014				
French speakers				- / 0.023		- / <0.001	+ / 0.042	
Occupation								
*Employed*				ref				
*In training*				- / 0.230				
*At home/unemployed*				+ / 0.004				
*Resident/annuitant*				+ / 0.867				
**Disease-related characteristics**								
Disease duration								
*0–5 years*	ref							
*5–15 years*	- / 0.137							
*>15 years*	- / 0.015							
Symptom severity	+ / <0.001	+ / <0.001	+ / <0.001	+ / 0.014	+ / <0.001	+ / <0.001	+ / <0.001	+ / 0.001
Highest treatment line								
*No treatment*	ref		ref		ref			
*5-ASA/steroids*	+ / 0.042		+ / 0.075		+ / 0.268			
*Immunomodulators*	+ / 0.020		+ / 0.024		+ / 0.189			
*Biologicals*	+ / 0.007		+ / 0.009		+ / 0.078			
History of resection surgery		+ / 0.026	- / 0.001					
History of extraintestinal manifestations						+ / 0.014		
**Psychosomatic characteristics**								
Signs of anxiety								
*None*	ref	ref		ref	ref	ref		ref
*Mild*	+ / 0.039	+ / <0.001		+ / 0.005	+ / 0.261	+ / 0.001		+ / 0.001
*Moderate*	+ / 0.061	+ / <0.001		+ / 0.005	+ / 0.007	+ / <0.001		+ / <0.001
*Severe*	+ / 0.047	+ / <0.001		+ / <0.001	+ / 0.018	+ / <0.001		+ / <0.001
Signs of depression								
*None*	ref						ref	
*Mild*	+ / 0.001						+ / <0.001	
*Moderate*	+ / 0.104						+ / 0.007	
*Severe*	+ / 0.441						+ / 0.543	
SF-36 MCS*					- / 0.043			
SF-36 PCS*					- / 0.003	- / <0.001		
IBDQ bowel* subscore					+ / 0.035			
IBDQ social* subscore		- / 0.015						- / <0.001
Task coping				+ / 0.002				
Emotional coping	+ / 0.025	+ / 0.005		+ / 0.006	+ / 0.013	+ / <0.001		+ / 0.015
Avoidance coping		- / 0.031						

* Component unrelated to anxiety and depression.

5-ASA, 5-aminosalicylic acid; MCS, Mental Component Summary; PCS, Physical Component Summary; IBDQ, Inflammatory Bowel Disease Questionnaire.

Women living in the French-speaking region of Switzerland had fewer concerns about symptoms or disease-related constraints and uncertainty than did those in the German-speaking region. Moreover, women at home or unemployed had higher concerns about disease-related constraints and uncertainty (p = 0.004). Socio-demographic characteristics did not have much effect on the level of concerns among men. French-speaking men had increased concerns about loss of body control than did German-speaking men (p = 0.042).

The burden linked to treatment was associated with concerns only for men. More specifically, socialization and stigmatization concerns significantly increased for men receiving immunomodulators (p = 0.020) or biologicals (p = 0.07); the same was observed for disease-related constraints and uncertainty (p = 0.024 and p = 0.009, respectively). A history of resection surgery was associated with lower concerns related to constraints and uncertainty among men (p = 0.001) and with higher socialization and stigmatization concerns among women (p = 0.026). The level of concern about symptoms was higher in women with a history of extraintestinal manifestations.

Increased levels of concern of any type were associated with signs of anxiety (p≤0.005) and higher emotional coping (p≤0.015) among women. Women’s concerns that were related to socialization and stigmatization and to loss of body control decreased with a better IBDQ social subscore and SF-36 Physical Component Summary score. Men’s concerns about socialization and stigmatization and about symptoms increased with signs of anxiety, although the significance of the association was not as high as that observed among women and was not present regardless of the level of anxiety. Increased concerns of men linked to socialization and stigmatization and to loss of body control were associated with signs of depression. Signs of depression were never associated with higher concerns in women. Symptom concerns decreased with better general QoL and with higher IBDQ bowel symptoms in men. Psychosomatic characteristics were not associated with concerns linked to symptoms among men.

## Discussion

This study aimed at assessing current and gender-specific concerns related to disease and treatments among IBD patients included in a national cohort study. Five of the 14 additional concerns we evaluated were among the top 10 scored by patients. Women in general had more concerns than did men. We found that signs of anxiety were highly associated with concerns, especially for women, and signs of depression were associated with only some types of concerns among men. General SF-36 and IBD-specific QoL measures were related to concerns related to symptoms. The burden linked to treatments only was associated with increased concerns among men. Symptom severity was the only factor highly associated with any type of concern and was unrelated to gender.

Our study was performed on 1102 patients included in a national cohort, which corresponds to the largest sample of patients screened for concerns at a country level.

We found that concern items traditionally used, based on the study conducted 25 years ago [5], have changed over time. Indeed, a set of new concerns were first expressed by patients during the qualitative study. Second, our cross-sectional survey showed that, when considering the whole sets of concerns, some of the old ones were rated much differently as observed in 1991 and 11 of the 14 new ones had a mean/median rating score > 40.

We observed that the mean rating scores of the most important concerns were higher than those measured in previous studies. This might indicate that IBD patients today have an increased consciousness about their disease and increased responsibility towards it. This positive aspect might also indicate an increased willingness to exchange and communicate about the disease. In comparison with previous studies, our study outlined different main concerns than those previously found. In our cohort, loss of bowel control and developing cancer were the highest concerns ranked by patients, in contrast to previously identified main concerns, i.e., ostomy bag, medication side effects[8,11,13,16], or uncertainty about the nature of the disease[13,16]. The concern about loss of bowel control had markedly increased between the 1990s [8] to 2000s (44.2–47.5)[11,13,16] and today (mean rating of 37.8 to 61.8). This might reflect the global burden of the disease and a change of attitude in patients, in that it seems easier to talk about IBD today than it was in the past. Such a mean score increase has also been observed for concerns linked to cancer (from 38.0[8] to 61.1). Increased patient information about their risk of cancer might explain why this concern scored higher than it did in previous studies[30]. It could also be due to increased penetrance of colon surveillance for cancer by repeated endoscopies. The effects of medications, of having an ostomy bag, and of the uncertain nature of the disease were still important concerns, as noted in previous studies. We also observed that six of the additional concerns we added were highly rated (mean rating scores above 55), even higher than any concerns explored in previous studies. Among those highly rated concerns, two were associated with symptoms: the link between stress and disease, and fatigue. Fatigue was shown to be associated with concerns in patients with IBD [31], and our study tends to further indicate that fatigue is not only associated with concerns and worries, but is a concern itself, especially for women. Patients included in the SIBDC mainly speak two national languages, German and French, as inclusions have not yet started in the Italian-speaking part of Switzerland. This is thus the first study in which a comparison of regional cultural aspects between two groups of patients evolving in the same healthcare system is possible, compared with the study of Levenstein et al.[13]. In our study, we found that some concerns were higher or lower according to language, with differences being observed in men and women.

Overall, we found that independent factors associated with concerns highly varied with gender, regardless of the factor, except symptom severity, which confirmed previous tendencies[15]. Symptom frequency was not associated with concerns. Socio-demographic variables were associated with constraints and uncertainty concerns, as previously found [11], but we confirmed this association only among women. Interestingly, the burden linked to treatments was associated with stigmatization and constraint concerns only among men, with men who were receiving biologicals being the most concerned about these issues. This might be partly explained because the highest proportion of men were employed, and thus men had more implicit difficulties in combining constraints linked to treatments and work. On the other hand–and because occupation status was not explicitly associated with these types of concerns in men–this might also indicate that men may have more difficulties in accepting, and thus sharing, disease-related disabilities at work or in other social groups. This might add to previous gender difference observations about crude but not adjusted group comparisons[12]. History of previous resection increased stigma concerns among women, but lowered constraint concerns among men, a more specific result than was previously found[9]. Another important finding was the association of concerns with psychosomatic measures, high differences depending on gender. In women, the greater the concerns, the higher were the signs of anxiety, regardless of the type of concern. Greater concerns related to socialization and stigmatization and loss of body control were associated with signs of depression in men, but not in women. This is in line with previous observations that men may be more emotionally affected than women when the disease is more complex and in the presence of associated complications[32]. Concerns about symptoms had a more important effect on men’s QoL than on that of women, as measured by standard general and disease-specific indicators. Two hypothesizes can be drawn; namely, the perception of QoL may be gender specific or symptoms may be perceived differently according to gender. A study by Hauser et al.[33] showed that women, after controlling for disease characteristics, had lower health-related QoL scores than did men. This may indicate that concerns related to symptoms may be able to better discriminate QoL measures in men, but not in women. Previous observations from qualitative studies indeed showed that women expressed a significantly lower level of general health-related QoL and more emotional disturbances related to their disease, as well as more frequent bowel symptoms compared with those of men[33]. Women seemed also to perceive a more negative impact and effects of IBD compared with those of men[33,34].

The main strength of our study was related to the large sample size of IBD patients that could be surveyed. Moreover, all of them could be deeply characterized because of the data that were collected through cohort enrollment and follow-up questionnaires. This comprised data from medical visits and from patient self-reported questionnaires, especially psychometric measures. In our study, we could investigate patients’ concerns at a country level. The fact that patients were followed in university centers or in private practices might help to minimize the potential selection bias, although our study is not population-based. One limitation was related to the survey’s response rate, which may lead to a nonresponse bias, although the impact on the results, in terms of potentially different distributions of concern ratings among nonresponders, is impossible to estimate. We could probably have slightly increased this rate by sending a reminder to patients, but the time available to conduct the study was limited and we decided not to bother patients too much by sending them questionnaires again in a short time frame. This also corresponds to the reality of long-term observational studies, with patients regularly invited to contribute to data collection[35], which may drive attrition.

In conclusion, worries and concerns of patients need to be considered when discussing perspectives about good quality of care. IBD is a high-burden disease, and patients seem to have important gender-specific [36] concerns related to their illness that need to be reassessed regularly, taking into account advances in healthcare over time.
